# Supplementary material for: The Gehan test identifies life-extending compounds overlooked by the log-rank test in the NIA Interventions Testing Program: Metformin, Enalapril, caffeic acid phenethyl ester, green tea extract, and 17-dimethylaminoethylamino-17-demethoxygeldanamycin hydrochloride
Source: GeroScience. 2024 Apr 17;46(5):4533–41. doi: 10.1007/s11357-024-01161-9 (PMC11335987; doi:10.1007/s11357-024-01161-9)
Supplement: Supplementary file 1 — Supplementary file1 (DOCX 395 KB) [file 11357_2024_1161_MOESM1_ESM.docx]

**Supplementary Table 1**

| **Conditions** | **Total** | **PH Not Violated** | **PH Violated** |
| --- | --- | --- | --- |
| **Log-rank, Sig; Gehan, Sig.** | 41 | 33 | 8 |
| **Log-rank, Sig; Gehan, Non-Sig.** | 4 | 4 | 0 |
| **Log-rank, Non-Sig; Gehan, Sig.** | 6 | 3 | 3 |
| **Log-rank, Non-Sig; Gehan, Non-Sig.** | 81 | 76 | 5 |
| **Total** | 132 | 116 | 16 |

**Table S1. Comparison of the log-rank and the Gehan tests stratified by Cox ZPH test.** The log-rank and Gehan tests are described in the Methods. The Cox ZPH test evaluates if the hazard ratio between drug treated and control mice is constant throughout the treatment period, and thus adheres to the proportional hazard assumption.

**Supplementary Table 2**

**Table S2. Summary of the results of the log-rank test, the Gehan test, and the Cox ZPH test for all interventions examined.** All drugs that are not significant in the log-rank test but significant (p < 0.05) in the Gehan test are marked in green. All the drugs that are significant (p < 0.05) in the log-rank test but not significant in the Gehan test are marked in orange.

**Supplementary Figure 1**

**Fig. S1, Survival curves and statistics for the other sex of the interventions newly identified by the Gehan test. (A)** Caffeic Acid Phenethyl Ester treated (CAPE, 300 ppm, from 4-month-old, n = 171) and control (n = 375) male mice. **(B)** Green Tea Extract treated (GTE, 2000 ppm, from 4-month-old, n = 145) and control (n = 276) male mice. **(C)** Metformin treated (1000 ppm, from 9-month-old, n = 140) and control (n = 281) female mice. **(D)** Enalapril treated (120 ppm, from 4-month-old, n = 139) and control (n = 289) female mice. **(E)** 17-dimethylaminoethylamino-17-demethoxygeldanamycin hydrochloride treated (DMAG, 30 ppm, from 6-month-old, n = 132) and control (n = 274) female mice. **(F)** 1,3-butanediol treated (BD, 100,000 ppm, from 6-month-old, n = 134) and control (n = 276) female mice.

**Supplementary Figure 2**

**Fig. S2,** **Survival curves for interventions that significantly increased survival according to the log-rank test but lost significance in the Gehan test.** **(A)** Acarbose treated (400 ppm, from 8 months, n = 139) and control (n = 287) female mice. **(B)** Acarbose treated (1000 ppm, from 4 months, n = 132) and control (n = 242) females. **(C)** (R/S)-1,3-butanediol treated (100000 ppm, from 6 months, n = 134) and control (n = 276) females. **(D)** Glycine treated (80000 ppm, from 9 months, n = 153) and control (n = 273) males.

**Supplementary Figure 3**

**Fig. S3, Survival curves for** **(A)** Enalapril treated (120 ppm, from 4-month-old, n = 170) and control (n = 357) male mice, and **(B)** Captopril treated (180 ppm, from 5-month-old, n = 150) and control (n = 285) male mice.
